# Odds and Ends

**Published:** 1870-04

**Authors:** E. Wildman


### Selections.

Odds and Ends.—By E. Wildman, M. D., D.D.S.—In the following medley, I propose, from time to time, to lay before our readers a series of formula and other matters that may be of practical use or of interest, as may occur to me or may be transcribed from my note book, without much regard to systematic arrangement, trusting that, at least, some of the younger members of our profession will find something therein of value to them. In doing so, I shall give recipes that have been tested and found good, not offering any others unless especially noted, and those well authenticated.

*Cements.*—Cements for retaining teeth to plate in fitting them down, or to try in the mouth before placing in the investment:

1. Gum Mastic, 8 parts.
   Yellow Wax, 4 parts.
   Color, q. s.
2. Gum Damar, 7 parts.
   Yellow Wax, 4 parts.
   Color, q. s.
3. Rosin, 2 parts.
   Wax, 1 part.

Nos. 1 and 2 possess very similar properties, being sufficiently adhesive and strong to answer the desired end, and are preferable to No. 3, on account of being firmer and more readily cleaned off the work, prior to applying borax. Gum damar being so much less expensive than mastic. I use No. 2.

To make these cements, place the vessel containing the wax and gum over a moderate heat, just sufficient to melt them, and stir until thoroughly incorporated; then add the color in quantity to produce the desired shade. Venetian red, drop lake or vermilion may be used. When all of the ingredients are well mixed, pour into a basin of cold water. To form into sticks, immerse the cake in water sufficiently warm to render it plastic. It is preferable to color it, as it renders it more sightly, and, also, we are better able to detect minute particles adhering to parts where solder is desired to flow and remove them. If desirable, it may be perfumed by adding an odoriferous oil just before pouring into cold water.

The following makes an adhesive cement of a dark color, which may be made more agreeable to the eye by the addition of venetian red or vermilion. It answers a good purpose to attach specimens to pedestals, &c.

4. Rosin, 4 parts.
Gutta Percha, 1 part.

First melt the rosin, then add the gutta percha, cut into shreds and stir until they are united.

No. 5 is a good water-proof cement, but does not possess much strength ; it will resist the action of water much better than shellac. An iron vessel coated with this composition will be protected from oxidation. In proof, I tested it on an iron frame aquarium which, after a constant exposure to water for four years, remained intact.

5. Pitch, 4 oz.

White Wax, 2½ oz.

Gutta Percha, 3½ oz.

First melt the pitch and wax together ; then add the gutta percha, cut into shreds, a little at a time, and stir until they are thoroughly incorporated.

To make a cement for building up pebble work, &c., for an aquarium, add to the above, after the ingredients are united, white clay, perfectly dry and finely pulverized, in quantity about one-fourth the weight of the mass. In using the pebbles or articles to be joined, they should be warm, and the cement, in a fluid state, applied with a brush to the surfaces to be united.

*Cap Cement*, (6,) so called by the late Professor Farraday. It makes a good strong cement to attach wood to glass. The parts to be united should be made quite hot, the cement applied in a fluid state, then firmly pressed together and retained until cool.

6. Rosin, 5 parts.
Yellow Wax, 1 part.
Venetian Red, 1 part.

The venetian red should be thoroughly dried and in a very fine powder, introduced a little at a time, and stirred into the melted mass.

*Shellac Cement.*—Gum shellac makes an excellent strong cement for joining small surfaces of wood together, and in many cases is far more convenient than glue. The shellac should be flowed upon the surfaces to be joined, and then they should immediately be pressed firmly together while the shellac is in a fluid state; in a minute or two the pieces will be found firmly united.

A convenient way of preparing gum shellac for laboratory use is to fuse the gum, as found in the shops, in a suitable vessel over a slow fire, being careful not to raise the heat higher than just sufficient to melt the gum, and, when fused, cast it in a mould; when cooled sufficiently to be plastic, but not adhesive, it may be worked into sticks. In manipulating this or No. 2, the hands should be kept moist with water.

*Alum Cement.*—This is principally useful to the dentist in securing an instrument to a pearl handle; it is strong and durable, when not exposed to moisture, and at the same time colorless.

Take the common alum crystals, place in a spoon over a quick fire; the alum melts in its water of crystallization so as to become perfectly fluid; while in this state, apply to the parts to be united and press together.

To produce a good result, the whole operation must be performed expeditiously, care to be observed not to allow the water of crystallization to be driven off, or the fluid to cool before the parts are joined.

*To Polish Ivory.*—Remove any scratches or file marks that may be present with finely pulverized pumice stone moistened with water. Then wash the ivory and polish with prepared chalk, applied moist upon a piece of chamois leather, rubbing quickly.

*To Polish Pearl.*—Take very finely pulverized rotten stone, and make into a thick paste by adding olive oil; then add sulphuric acid, (oil of vitriol,) a sufficient quantity to make into a thin paste.

This is to be applied on a velvet cork; rub quickly, and as soon as the pearl takes the polish wash it. This mixture when properly applied, will give to pearl a brilliant polish.

